# Carcinome épidermoïde du bassinet découvert par un envahissement pariétal et cutané: une présentation inhabituelle

**DOI:** 10.11604/pamj.2018.31.246.10266

**Published:** 2018-12-26

**Authors:** Maher Slimane, Manel Hadidane, Hatem Bouzaiene, Maha Driss, Olfa Jaidane, Houda Henchiri, Monia Hechiche, Khaled Rahal

**Affiliations:** 1 Service de Chirurgie Carcinologique, Institut Salah Azaiez, Boulevard 9 Avril 1938, Bab Saadoun, 1007 Tunis, Faculté de Médecine de Tunis El Manar II, 15 rue Djebel Lakhdhar, La Rabta, Tunisie; 2 Service d 'Anatomopathologie, Institut Salah Azaiez, Boulevard 9 Avril 1938, Bab Saadoun, 1007 Tunis, Faculté de Médecine de Tunis El Manar II, 15 rue Djebel Lakhdhar, La Rabta, Tunisie

**Keywords:** Carcinome épidermoïde, bassinet, envahissement cutané, Squamous cell carcinoma, renal pelvis, skin invasion

## Abstract

A travers cette étude clinique nous présentons un cas rare de carcinome épidermoïde du bassinet avec envahissement cutané de la paroi abdominale chez une patiente aux antécédents d'infections urinaires hautes à répétition sur des calculs rénaux. Le motif de consultation était la découverte d'une lésion cutanée lombaire droite. L'uro-scanner montrait une masse rénale droite étendue aux parties molles adjacentes dont la biopsie a révélé un carcinome épidermoïde du bassinet. Après une revue de la littérature, c'est le premier cas d'un carcinome épidermoïde du bassinet découvert par un envahissement cutané.

## Abstract (en)

We here report a rare case of squamous cells carcinoma of the renal pelvis with abdominal wall skin invasion in a patient with a history of recurrent upper urinary tract infections due to kidney stones. A right lumbar skin lesion was the reason for consultation. Uroscanner showed right renal mass extended to the adjacent soft tissues. Biopsy showed squamous cell carcinoma of the renal pelvis. After having conducted a literature review we can say that this is the first case of squamous cell carcinoma of the renal pelvis detected due to skin invasion.

## Introduction

Le carcinome épidermoïde du bassinet est une affection grave et rare. Il représente 0,5 à 0,8% des tumeurs rénales malignes et 0,5 à 8% des tumeurs du tractus urinaire supérieur [1, 2]. Le carcinome épidermoïde du bassinet survient le plus souvent après un long passé de lithiase rénale et d'infections urinaires à répétition non ou mal traitées. Il représente 9 à 17% des tumeurs pyélocalicielles primitives [3]. Il constitue un diagnostic différentiel avec le carcinome urothélial [4]. Le principal facteur favorisant est la présence de calculs associés à une infection chronique avec un risque relatif de 2,5 [1, 5, 6]. Un antécédent de calcul rénal ou de chirurgie pour calcul rénal a été retrouvé dans 12,3% des cas dans une série et jusqu’à 100% des cas dans une autre série [1, 2]. Selon Li, l’incidence du carcinome épidermoïde du rein est estimée à 2% des patients porteurs de lithiase rénale récidivante [1]. Au même stade, le carcinome urothélial a le même pronostic que le carcinome épidermoïde. A la différence du carcinome urothélial, le carcinome épidermoïde est plus fréquemment découvert à des stades avancés [7]. L'envahissement cutané à travers la paroi abdominale est exceptionnel. Giannopoulos A et *al.* ont rapporté en 1989 un cas où le carcinome a été révélé par une fistule cutanée secondaire à une pyonéphrose [8]. D'après notre recherche bibliographique, cet envahissement cutané révélateur du carcinome épidermoïde du bassinet a peu été rapporté dans la littérature d'où l'originalité de notre cas.

## Patient et observation

**Figure 1:**
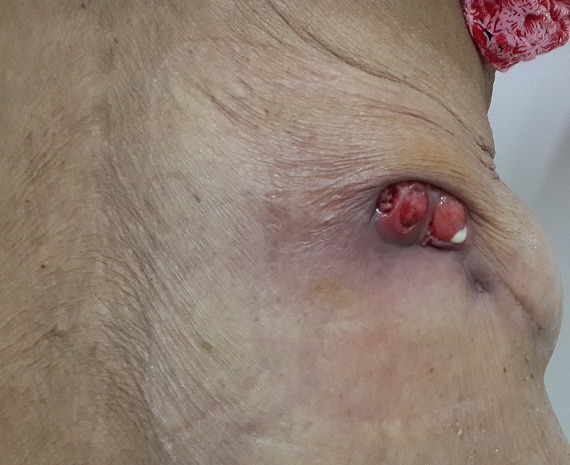
envahissement cutané ulcéro-bourgeonnant de la tumeur rénale droite

**Figure 2:**
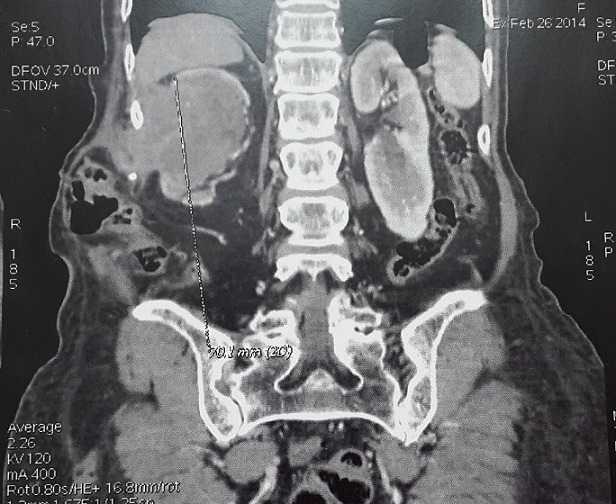
uro-scanner coupe sagittale et frontale, masse rénale droite hétérogène peu rehaussée par le produit de contraste infiltrant l'espace péri-rénal avec effraction capsulaire et extension au niveau des parties molles en regard

**Figure 3:**
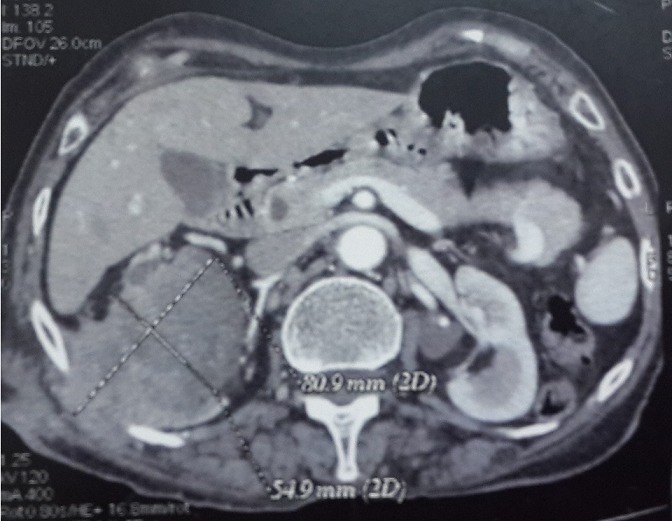
pièce de néphrectomie élargie emportant la dernière côte flottante et la paroi antéro latérale droite en regard

Il s'agit de Madame X âgée de 76ans, non tabagique, hypertendue, traitée à plusieurs reprises pour des infections urinaires hautes sur calculs caliciels et ayant eu une pyélolombotomie percutanée il y a 2 ans. La patiente a consulté pour une masse lombaire droite douloureuse. La patiente ne rapportait pas de signes urinaires, particulièrement pas d'hématurie. L'examen clinique trouvait une masse ulcéro-bourgeonnante de la fosse lombaire droite de 30x40mm (Figure 1). La palpation abdominale a trouvé une masse lombaire droite fixée de 100x70mm qui était en continuité avec la masse cutanée. Le scanner thoraco-abdomino-pelvien a montré une masse médio- rénale droite tissulaire, hétérogène de 93x54x80 mm se développant à la fois au sein du pyélon qui était distendu et au niveau du cortex rénal avec un important franchissement capsulaire et une extension tumorale dans les parties molles de la paroi abdominale postéro-latérale. Cette masse envahissait le 11^ème^ espace intercostal et le plan musculaire sous-jacent. La veine rénale droite était perméable. L'étage thoracique était sans anomalie. L'uro-scanner a objectivé en plus de la masse médio rénale droite un rein droit de taille diminuée, mesurant 90cm de grand axe, à corticale amincie ayant un retard sécrétoire et surtout excrétoire siège de plusieurs calcifications corticales polaires inférieures et d'une importante dilatation pyélo-calicielle hétérogène spontanément hyperdense et non rehaussée par le produit de contraste en amont d'un rétrécissement sous-pyélique sans obstacle lithiasique visible. Il s'y associait plusieurs adénopathies hypodenses rétro-caves, latéro-aortiques et inter aortico-caves (Figure 2, Figure 3). Le rein gauche était sans anomalies. L'examen anatomopathologique d'une biopsie scanno-guidée de la masse rénale a révélé une prolifération tumorale maligne prenant naissance à partir du revêtement de surface qui était malpighien métaplasique. Cette prolifération infiltrait le chorion sous forme d'amas et de massifs de cellules polygonales jointives qui s'enroulaient et élaboraient de la kératine sous forme de globes cornés. Les noyaux étaient ovoïdes, volumineux, atypiques, hyper chromatiques et fortement nucléolés. De nombreuses figures de mitoses étaient observées. Le tout était en faveur d'un carcinome épidermoïde bien différencié et kératinisant.

**Figure 4:**
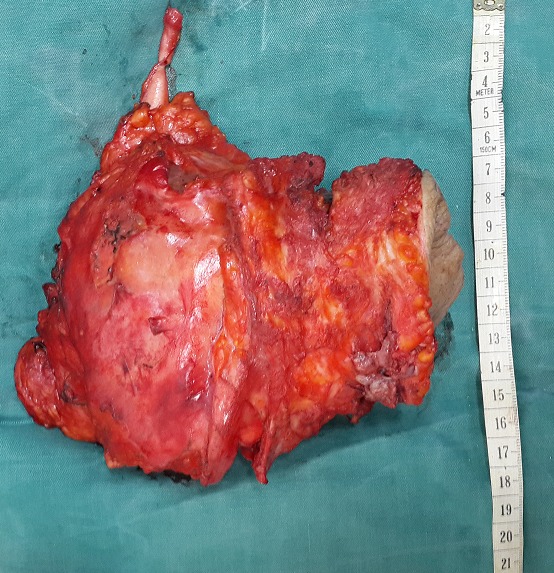
glomérules rénaux normaux adjacents à des foyers de carcinome épidermoïde. Hématoxiline Eosine (HE), agrandissement x 100

La fonction rénale a été évaluée par un dosage de la créatininémie à 70µmol/l soit une clearance calculée selon la formule de Cockcroft de 51 ml/min. Il existait une anémie à 8 g/dl hypochrome microcytaire sans syndrome inflammatoire biologique. La patiente a été opérée par double voie médiane et lombaire droite. L'intervention a débuté par une laparotomie médiane. A l'exploration, le rein droit était pris dans une masse tumorale blanchâtre, friable mesurant 150x150mm envahissant la paroi postérolatérale droite jusqu'à la peau. Il n'y avait pas de métastases hépatiques ni de carcinose péritonéale. Le premier temps opératoire était de libérer la masse tumorale de la face inférieure du foie avec décollement colo-pariétal droit, et libération du colon ascendant et transverse. Un décollement de Kocher était nécessaire pour exposer la face antérieure de la veine cave. La dissection s'était poursuivie par la ligature section de l'artère et de la veine rénale droites pour permettre un décollement médian et postérieur de la masse rénale. Après avoir libéré du pôle inférieur du rein, on a sectionné l'uretère à 6cm du pyélon. Devant le caractère fixe de la partie postéro-externe de la masse à la paroi (la tumeur envahissait tous les muscles de la paroi postéro-latérale ainsi que les 2 dernières côtes flottantes), nous avons opté pour un deuxième abord de la tumeur. Le deuxième temps opératoire consistait en une incision losangique circonscrivant la lésion cutanée sur la paroi postéro-latérale droite. La dissection a débuté par la section des tissus sous-cutanés, musculaires et les 2 dernières côtes flottantes. Ceci nous a permis de rejoindre le plan de dissection par voie médiane. Une fois totalement libérée, la masse tumorale a été réséquée en monobloc en emportant ainsi, le rein et son pédicule, la surrénale, 6 centimètre d'uretère proximal, les 2 dernières côtes flottantes droites et la paroi cutanéo-musculaire envahie (Figure 4). La perte de substance pariétale a été recouverte par un lambeau musculaire de rotation utilisant le muscle oblique externe en gardant sa vascularisation supérieure.

**Figure 5:**
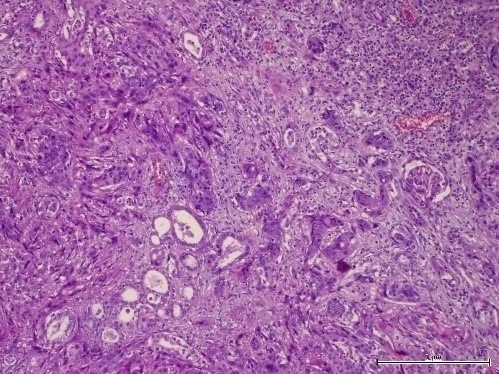
uro-scanner coupe sagittale et frontale, masse rénale droite hétérogène peu rehaussée par le produit de contraste infiltrant l'espace péri-rénal avec effraction capsulaire et extension au niveau des parties molles en regard

Les suites opératoires ont été simples avec un lambeau de bonne qualité. L'examen anatomopathologique macroscopique définitif montrait un rein envahi par une tumeur blanchâtre friable détruisant tout le rein et s'étendant dans la graisse péri-rénale et les cavités pyélo-calicielles qui étaient dilatées et kystiques. A l'histologie, la tumeur était faite d'une prolifération de cellules de type malpighien agencées en lobules et travées centrés par de nombreuses squames de kératine. Les atypies cytonucléaires étaient marquées. Les limites cutanées, osseuses et urétérales chirurgicales étaient saines (Figure 5). La patiente n'a pas eu de traitement adjuvant. Pendant 1 an et demi après la chirurgie, la patiente était régulièrement suivie à notre consultation avec un examen clinique, une échographie abdominale et une radio du thorax tous les 6 mois, sans signes de récidives. Après un recul de 18 mois, la patiente se portait bien.

## Discussion

Comme c'est le cas de notre patiente, les infections urinaires à répétition sur des lithiases rénales constituent un facteur de risque important des carcinomes épidermoïdes du bassinet. Le tabac peut être aussi un facteur de risque comme pour les localisations vésicales mais ce n'est pas le cas de notre malade. La Bilharziose a été rapportée comme un facteur de risque pour les localisations urinaires des carcinomes épidermoïdes au niveau vésical et urétral principalement [9]. L'âge moyen de survenu est situé entre 56 et 69 ans [10, 11] avec une prédominance masculine [9, 11]. La symptomatologie est constituée principalement d'hématurie dans 60% des cas [11] et de douleurs lombaires, qui perdent leur valeur d'alarme puisqu'elles sont souvent associées aux épisodes infectieux antérieurs [12]. Les signes infectieux souvent présents, peuvent aussi nous éloigner du diagnostic de carcinome, d'autant plus que les ECBU (examen cytobactériologique des urines) sont souvent positifs. La cytologie urinaire peut affirmer le diagnostic de carcinome épidermoïde [4]. L'extension de la tumeur à la paroi est un signe important, certes tardif, pour nous alarmer. La particularité de notre cas clinique est que l'envahissement pariétal, déjà décrit dans les autres cas de la littérature, a été dépassé avec un envahissement de la peau, très peu rapporté dans la littérature. De plus, c'est le principal motif de consultation de notre patiente. Sur le plan radiologique, la présence de calculs ne doit pas faire égarer le diagnostic. L'association sur le scanner ou l'échographie de calcul rénal, de lacune pyélocalicielle et ou de syndrome de masse rénal doit nous alarmer d'autant plus qu'il s'agit de calculs anciens [5]. Une étude rétrospective étudiant les aspects radiologiques du carcinome épidermoïde du rein a montré que les images radiologiques de lésions obstructives, de lacunes, ou de rein non fonctionnel sur l'urographie intraveineuse n'étaient pas spécifiques, ce qui tardait encore plus le diagnostic [13]. Le scanner garde une place importante pour le diagnostic et le bilan d'extension locorégional et à distance. Il permet également une biopsie radioguidée. Histologiquement, il existe une différenciation malpighienne dans toute la tumeur, contrairement aux inflexions épidermoïdes fréquemment observées dans les carcinomes urothéliaux [14]. Une composante épidermoïde intraépithéliale est en faveur du caractère primitif de la tumeur [10, 11]. La tumeur est caractérisée par une extension principalement locale [1]. A la différence des autres carcinomes urothéliaux, il n'y a pas de dissémination par voie urétérale [9]. Les métastases ganglionnaires sont moins fréquentes, elles sont plutôt osseuses, pulmonaires ou hépatiques [11].

La prise en charge thérapeutique de ces cancers devrait prendre en considération l'âge, l'état général et le choix du patient ainsi que le stade et le grade de la tumeur [15]. La principale chirurgie à visée curative consiste en une néphrectomie élargie avec urétérectomie totale emportant une collerette vésicale autour de l'orifice urétérale [15]. Chez notre patiente, une chirurgie radicale ne pouvait être réalisée que par 2 abords différents de la tumeur à savoir par voie médiane par voie et lombaire. Ainsi, les limites chirurgicales étaient saines et la masse tumorale et son envahissement pariétal étaient emportés en monobloc. Cependant, une urétérectomie totale avec une collerette vésicale aurait dû être réalisée. La chimiothérapie, l'immunothérapie et la radiothérapie sont principalement indiquées dans les stades avancées inopérables avec des effets limités sur la survie [16]. La pratique d'un curage ganglionnaire a peu d'arguments devant une tumeur localement avancée avec un pronostic défavorable. Le pronostic de ces tumeurs reste cependant mauvais avec une survie moyenne de 07 mois et une survie à 5 ans ne dépassant pas les 10% [1-15]. Avec ce type de cancer, la prévention prend toute son importance. Il est primordial de traiter les calculs rénaux correctement et prévenir les infections urinaires hautes à répétition, principal facteur de risque du carcinome épidermoïde du bassinet. Dans notre cas, on peut supposer que la pyélolombotomie percutanée avait un rôle pour favoriser l'extension de la tumeur jusqu'à la peau. En effet d'autres cas devraient être rapportés pour étudier le risque de la pyélolobotomie percutanée.

## Conclusion

Le mauvais pronostic de cette entité peut être expliqué par le retard diagnostic et de prise en charge de ces cancers, plutôt qu'une agressivité plus importante comme pour les tumeurs urothéliales. Concernant notre patiente, la chirurgie, lourde soit-elle, était la meilleure option. Il est vrai que dans un premier temps, nous étions réticents à la chirurgie devant l'âge et l'état général de la patiente. Mais ceci nous a permis de traiter notre malade et soulager ses douleurs. La chirurgie est la meilleure option thérapeutique pour cette entité. Les traitements systémiques et les thérapies ciblées restent une option pour les cas inopérables.

## Conflits d’intérêts

Les auteurs ne déclarent aucun conflit d'intérêts.
